# Gender related outcome differences in off-pump coronary artery bypass grafting

**DOI:** 10.3389/fcvm.2025.1641784

**Published:** 2025-08-01

**Authors:** Riswan Akram, Friedrich Sobik, Tim Knochenhauer, Sebastian A. Philipp, Jens Brickwedel, Xiaoqin Hua, Beate Reiter, Svante Zipfel, Yvonne Schneeberger, Evaldas Girdauskas, Hermann Reichenspurner, Bjoern Sill, Andreas Schaefer

**Affiliations:** ^1^ Department of Cardiology, Elbe Clinic Stade, Stade, Germany; ^2^ Department of Cardiovascular Surgery, University Heart and Vascular Center Hamburg, Hamburg, Germany; ^3^ Department of Cardiac Surgery, University Hospital Cologne, Cologne, Germany; ^4^ Department of Cardiac Surgery, Asklepios Clinic St. Georg, Hamburg, Germany; ^5^ Department of Cardiothoracic Surgery, Augsburg University Medical Centre, Augsburg, Germany

**Keywords:** coronary artery bypass grafting, coronary artery disease, gender, STEMI, NSTEMI, OPCAB

## Abstract

**Objectives:**

Surgical myocardial revascularization shows impaired outcomes in women compared to men. Investigation of gender related outcome differences comprises of different operative strategies potentially hampering interpretation of data. We herein aimed to investigate gender related outcome differences in off-pump coronary artery bypass grafting (OPCAB) only.

**Methods:**

Between 2016 and 2021, 1,075 consecutive patients underwent OPCAB at our center. Of those 880/1,075 were male (81.9%) and 195/1,075 were female (18.1%). Kaplan–Meier analysis was used for investigating differences in survival probabilities. Identification of risk factors was conducted by logistic regression.

**Results:**

Male patients showed a higher rate of reduced LVEF < 35% (88/880, 10% vs. 9/195, 4.61%; *p* = 0.025) and impaired renal function (creatinine: 1.17 ± 0.76 vs. 1.03 ± 0.59; *p* = 0.016). In female patients less utilization of both internal mammary arteries was documented (502/880, 57.04% vs. 74/195, 37.94%; *p* < 0.001). Procedure time (256.13 min vs. 238.02 min; *p* < 0.001) and number of distal anastomoses (2.40 ± 0.83 vs. 2.11 ± 0.82; *p* < 0.001) were lower in female patients. 30-day mortality (16/880, 0.34% vs. 4/195, 0.51%; *p* = 0.77) and rates of disabling stroke (3/880, 1.81% vs. 1/195, 2.05%; *p* = 0.55) were similar between groups. In logistic regression analysis age (OR 1.079; CI 1.001- 1.162; *p* = 0.047) and impaired renal function (OR 1.495; CI 1.090–2.051; *p* = 0.013) were identified as independent risk factors for 30-day mortality.

**Conclusions:**

Male and female patients present similar 30-day outcomes after OPCAB suggesting a potential benefit of OPCAB in female patients. However, female patients receive more saphenous vein grafts compared to men, which may lead to impaired long-term outcomes.

## Introduction

Coronary artery bypass grafting (CABG) is the most commonly performed cardiac surgery worldwide, recommended for treating complex coronary artery disease (CAD) in patients with intermediate to high SYNTAX (Synergy between Percutaneous Coronary Intervention with TAXUS and Cardiac Surgery) score and diabetes (1, 2). However, only 20%–30% of patients undergoing CABG are women (3, 4). Several studies report worse outcomes in women after CABG, including higher incidences of acute mortality, stroke, and postoperative myocardial infarction (5–7).

Reasons for these outcome differences are likely multifactorial including delayed diagnosis in women due to atypical symptoms, leading to later presentation for CABG (8, 9), a higher comorbidity burden and more urgent presentation at the time of surgery in women (10, 11), a higher incidence of non-obstructive CAD (12), smaller coronary arteries more prone to spasm (13, 14), and less frequent complete revascularization, with increased use of unfavorable bypass grafts (15, 16).

Most studies and registries investigating gender-related outcome differences in CABG combine various operative strategies, including on-pump CABG, OPCAB, and beating heart CABG. Therefore, interpretation of these findings is challenging. Previously gender-related outcome differences in on-pump CABG were described, with higher rates of emergency procedures, use of saphenous vein grafts (SVG), and postoperative complications like myocardial infarction, stroke, and wound healing disorders (WHD) as well as increased 30-day mortality in women (13).

To further clarify the impact of surgical strategies in CABG on gender-specific outcomes, our current study aims to specifically investigate gender-related differences in OPCAB only.

This work is part of the first author's dissertation thesis conducted at the University Medical Center Hamburg Eppendorf.

## Patients and methods

### Ethical statement

Data acquisition was performed anonymized and retrospectively. Therefore, in accordance with German law, no ethical approval is needed and informed patient consent was waived.

### Patients and definitions

Between 01/2016 and 12/2021, 1,075 consecutive patients underwent isolated OPCAB at our center. Of those 880/1,075 were male (81.9%, group 1) and 195/1,075 were female (18.1%, group 2). Assignment to gender followed biological sex.

Primary endpoints for this study are adverse events during 30 days of the index procedure including all-cause mortality, postoperative myocardial infarction, major stroke and acute renal failure [Acute kidney injury network (AKIN) III]. Secondary outcomes include resternotomy for bleeding, wound healing disorders (WHD), sepsis, New York Heart association (NYHA) functional class ≥3 and postoperative creatinine levels.

Complete revascularization was defined as revascularization of all coronary segments with a stenosis of ≥50% supplying viable myocardium (17). Wound healing disorders were defined as postoperative infection involving the sternum and mediastinal space or isolated infection of the sternal subcutaneous layer. After surgery and completion of hospital stay patients were referred to cardiac rehabilitation as standard of care.

### Statistical analyses

Continuous variables are reported as mean ± standard deviation. Categorical variables are presented as proportions. Baseline differences between male and female patients undergoing OPCAB were detected using the chi^2^-test, the Fisher's exact test and the t-test. Non-parametric data was analysed using the Mann–Whitney-test. For validation of normal data distribution, the Kolmogorow-Smirnow-test was utilized. Kaplan–Meier analysis was implemented for investigating differences in survival probabilities between male and female patients after OPCAB. Identification of independent risk factors for 30-day mortality was conducted by logistic regression including the variables sex, age and preoperative left ventricular ejection fraction (LVEF) < 35%.

All test were two tailed and *p* < 0.05 was considered statistically significant. All statistical analyses were performed using the statistical software SPSS (IBM SPSS Statistics for Windows, Version 27.0. IBM Corp., Armonk, NY, USA).

## Results

### Baseline demographics

Male (group 1) and female patients (group 2) undergoing OPCAB presented no significant differences in baseline parameters regarding age (group 1: 68.99 ± 9.91 vs. group 2: 69.91 ± 9.94 years; *p* = 0.2413) and comorbidity and symptom burden (extracardiac artheropathy: 198/880, 22.5% vs. 42/195, 21.5%, *p* = 0.8531; NYHA functional class ≥ III: 469/880; 53.29% vs. 115/195, 58.97; *p* = 0.4683). However, common risk stratification tools (European System for Cardiac Operative Risk Evaluation II: 2.09% vs. 2.69%; *p* = 0.001) indicated a higher perioperative risk in group 2. Male patients presented with higher baseline creatinine levels (1.17 ± 0.76 vs. 1.03 ± 0.59 mg/dl; *p* = 0.0159) and a higher number of diseased coronary vessels (2.44 ± 1.03 vs. 2.28 ± 0.41; *p* = 0.033). Female patients presented more often with preoperative non ST-elevation infarction (NSTEMI) (216/880, 24.54% vs. 50/195, 25.64%; *p* = 0.566). Furthermore, the male population showed higher rates of LV dysfunction (LVEF < 35%: 88/880, 10.0% vs. 9/195, 4.61%; *p* = 0.025).

Detailed patient demographics are summarized in Table 1.

**Table 1:** Baseline data of male and female patients undergoing OPCAB.

	Male (*n* = 880)	Female (*n* = 195)	*p*-value
Age, years	68.99 ± 9.91	69.91 ± 9.94	0.24
BMI, kg/m^2^	28.28 ± 7.29	28.04 ± 5.43	0.66
Prior CABG, *n* (%)	9 (1.02)	1 (0.51)	1.00
Prior PCI, *n* (%)	174 (19.77)	29 (14.87)	0.22
Myocardial infarction^a^, *n* (%)	249 (28.29)	60 (30.76)	0.61
STEMI	33	10	0.42
NSTEMI	216	50	0.56
COPD^b^, *n* (%)	65 (7.38)	18 (9.23)	0.46
Prior stroke, *n* (%)	101 (11.47)	18 (9.23)	0.45
Extracardiac atheropathy^b^, *n* (%)	198 (22.50)	42 (21.53)	0.85
LVEF ≤35%, *n* (%)	88 (10)	9 (4.61)	0.02
Diabetes, *n* (%)	71 (8.06)	23 (11.79)	0.13
Dialysis, *n* (%)	13 (1.47)	5 (2.56)	0.35
Creatinine, mg/dl	1.17 ± 0.76	1.03 ± 0.59	0.01
Number of diseased vessels	2.44 ± 1.03	2.28 ± 0.41	0.03
One vessel disease, *n* (%)	64 (7.3)	29 (14.9)	0.001
Two vessel disease, *n* (%)	176 (20.0)	40 (20.5)	0.84
Three vessel disease, *n* (%)	640 (72.7)	126 (64.6)	0.02
EuroSCORE II, % median	2.09 ± 2.35	2.69 ± 2.55	0.001
NYHA ≥ III, *n* (%)	469 (53.29)	115 (58.97)	0.46

OPCAB, off-pump coronary artery bypass; BMI, body mass index; PCI, percutaneous coronary intervention; STEMI, ST-elevation myocardial infarction; NSTEMI, non ST-elevation myocardial infarction; COPD, chronic obstructive pulmonary disease; LVEF, left ventricular ejection fraction; EuroSCORE II, European System for Cardiac Operative Risk Evaluation II; NYHA, New York Heart Association.

^a^ During 30 days prior to CABG.

^b^ According to EuroSCORE definitions.

### Periprocedural data

No differences were seen in rates of OPCAB as emergency procedure (40/880, 4.55% vs. 11/195, 5.64%; *p* = 0.577). Procedure time was lower in female patients (256.13 vs. 238.02 min; *p* = 0.0001). Accordingly, number of performed distal bypass anastomoses was lower in group 2 (2.4 ± 0.8 vs. 2.1 ± 0.8; *p* < 0.001). Utilization of bilateral internal mammary artery (BIMA) was more frequently conducted in male patients (502/880, 57.04% vs. 74/195, 37.94%; *p* = 0.0052), whereas graft choice strategies including combination of the left internal mammary artery (LIMA) and saphenous vein grafts (SVG) (220/880, 25% vs. 66/195, 33.84%; *p* = 0.076) or single IMA (131/880, 14.88% vs. 50/195, 25.64%; *p* = 0.004) were more often applied in female patients. Accordingly, incidence of one vessel disease was higher in female patients. No differences between groups were found regarding rates of complete revascularization.

In group 2 a higher rate of red blood cell unit (RBC) administration (188/880 vs. 83/195; *p* = 0.0001) and a higher number of administered RBC (0.58 ± 1.76 vs. 1.03 ± 1.63 *p* = 0.001) was documented.

Detailed periprocedural data are summarized in Table 2.

**Table 2:** Periprocedural data of male and female patients undergoing OPCAB.

	Male (*n* = 880)	Female (*n* = 195)	*p*-value
Urgency, *n* (%)			
Elective	840 (95.45)	184 (94.36)	0.95
Urgent	40 (4.55)	11 (5.64)	0.57
Procedure time, min median	256.13	238.02	0.0001
Number of bypasses, *n*	2.40 ± 0.83	2.11 ± 0.82	0.0001
Bypass graft strategy			
BIMA grafting, *n* (%)	502 (57.04)	74 (37.94)	0.005
LIMA + SVG, *n* (%)	220 (25)	66 (33.84)	0.07
SVG, *n* (%)	4 (0.45)	1 (0.51)	1
IMA + radialis, *n* (%)	10 (1.13)	3 (1.53)	0.71
Single IMA, *n* (%)	131 (14.88)	50 (25.64)	0.004
Complete revascularization, *n* (%)	654 (74.31)	141 (72.30)	0.85
Prolonged inotropes ≥24 h, *n* (%)	18 (2.04)	6 (3.07)	0.42
Conversion to ECC, *n* (%)	6 (0.68)	1 (0.51)	1
RBC administration, *n* (%)	188 (21.4)	83 (42.6)	0.0001
Numbers of RBC	0.58 ± 1.76	1.03 ± 1.63	0.001

OPCAB, off-pump coronary artery bypass; BIMA, bilateral internal mammary artery; LIMA, left internal mammary artery; SVG, sapheneous vein graft; ECC, extracorporeal circulation; RBC, packed red blood cells.

### 30-day outcomes

No significant difference regarding 30-day mortality was seen (16/880, 1.81% vs. 4/195, 2.05%; *p* = 0.77) between groups. Postoperative complications showed similar rates between groups regarding wound healing disorders (17/880, 1.93% vs. 7/195, 3.58%; *p* = 0.12) and resternotomy (10/880, 1.13% vs. 2/195, 1.02%; *p* = 1.0). Rates of postoperative coronary angiography were similar between groups (30/880, 3.40% vs. 8/195, 4.10%; *p* = 0.66). Mean ventilation time (9.62 ± 36.15 vs. 8.35 ± 15.72 h; *p* = 0.63), and rates of prolonged ventilation time >24 h (23/880, 2.61% vs. 5/195, 2.56%; *p* = 1.0) presented no differences between groups. Intensive care unit stay time was similar between groups (2.45 ± 2.91 vs. 2.37 ± 1.87 days; *p* = 0.08), as well as hospital stay time (8.29 ± 4.00 vs. 8.89 ± 5.99 days; *p* = 0.08). Rate of myocardial infarction during 30 days after the procedure was similar between groups (20/880, 2.27% vs. 5/195, 2.56%; *p* = 0.80), as well as rates of disabling stroke (3/880, 0.34% vs. 1/195, 0.51%; *p* = 0.55) and rates of renal failure (AKIN III) (11/880, 1.25% vs. 4/195, 2.05%; *p* = 0.49).

**Figure 1:**
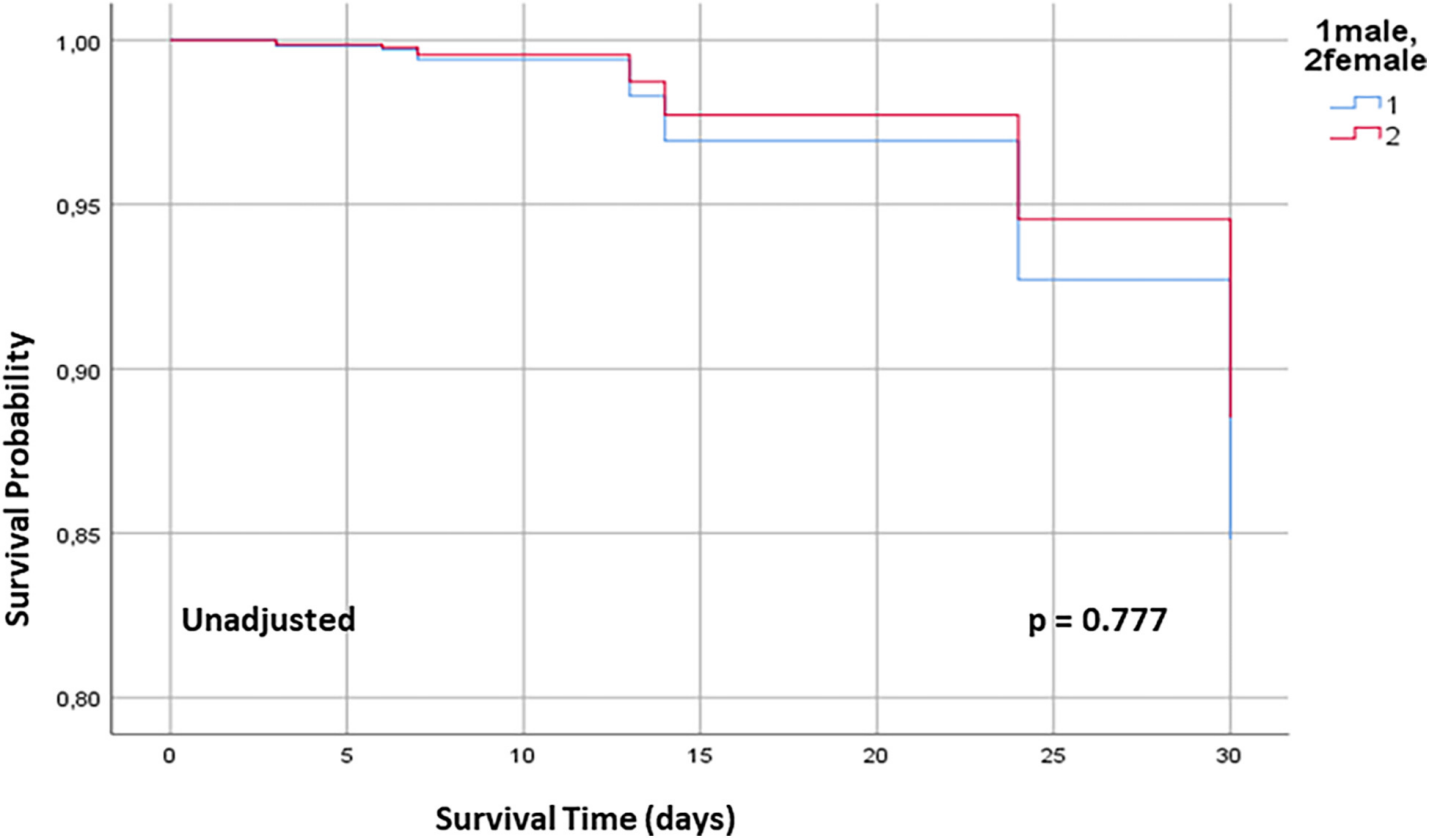
30 days Kaplan–Meier survival curve for male and female patients undergoing OPCAB. OPCAB off-pump coronary artery bypass.

Detailed 30-day outcome parameters are summarized in Table 3. See Figure 1 for Kaplan–Meier curve.

**Table 3:** 30-day outcome parameter of male and female patients undergoing OPCAB.

	Male (*n* = 880)	Female (*n* = 195)	*p*-value
Mortality, *n* (%)	16 (1.81)	4 (2.05)	0.77
WHD^a^, *n* (%)	17 (1.93)	7 (3.58)	0.18
Resternotomy, *n* (%)	10 (1.13)	2 (1.02)	1
Coronary angiography, *n* (%)	30 (3.40)	8 (4.10)	0.66
PCI, *n* (%)	8 (0.91)	2 (1.03)	1.0
Ventilation time, h	9.62 ± 36.15	8.35 ± 15.72	0.63
Ventilation >24 h, *n* (%)	23 (2.61)	5 (2.56)	1
ICU stay, days	2.45 ± 2.91	2.37 ± 1.87	0.08
Hospital stay, days	8.29 ± 4.00	8.89 ± 5.99	0.08
Myocardial infarction, *n* (%)	20 (2.27)	5 (2.56)	0.79
Disabling stroke, *n* (%)	3 (0.34)	1 (0.51)	0.55
Renal failure (AKIN III), *n* (%)	11 (1.25)	4 (2.05)	0.49

OPCAB, off-pump coronary artery bypass; WHD, wound healing disorders, PCI, percutaneous coronary intervention; ICU, intensive care unit; AKIN, acute kidney injury network; MACE, major adverse cardiac even.

^a^ Including superficial wound healing disorders and deep sternal wound infection.

In logistic regression analysis independent risk factors for 30-day mortality consisted of increased preoperative creatinine levels (OR 1.495; CI 1.090–2.051; *p* = 0.013) and age (OR 1.079; CI 1.001–1.162; *p* = 0.047). Preoperative LVEF <35% (OR 1.029; CI 0.227–4.675; *p* = 0.970) and gender (OR 1.084, CI 0.345–3.404; *p* = 0.89) were not predictive for mortality during 30 days after OPCAB.

**Figure 2:**
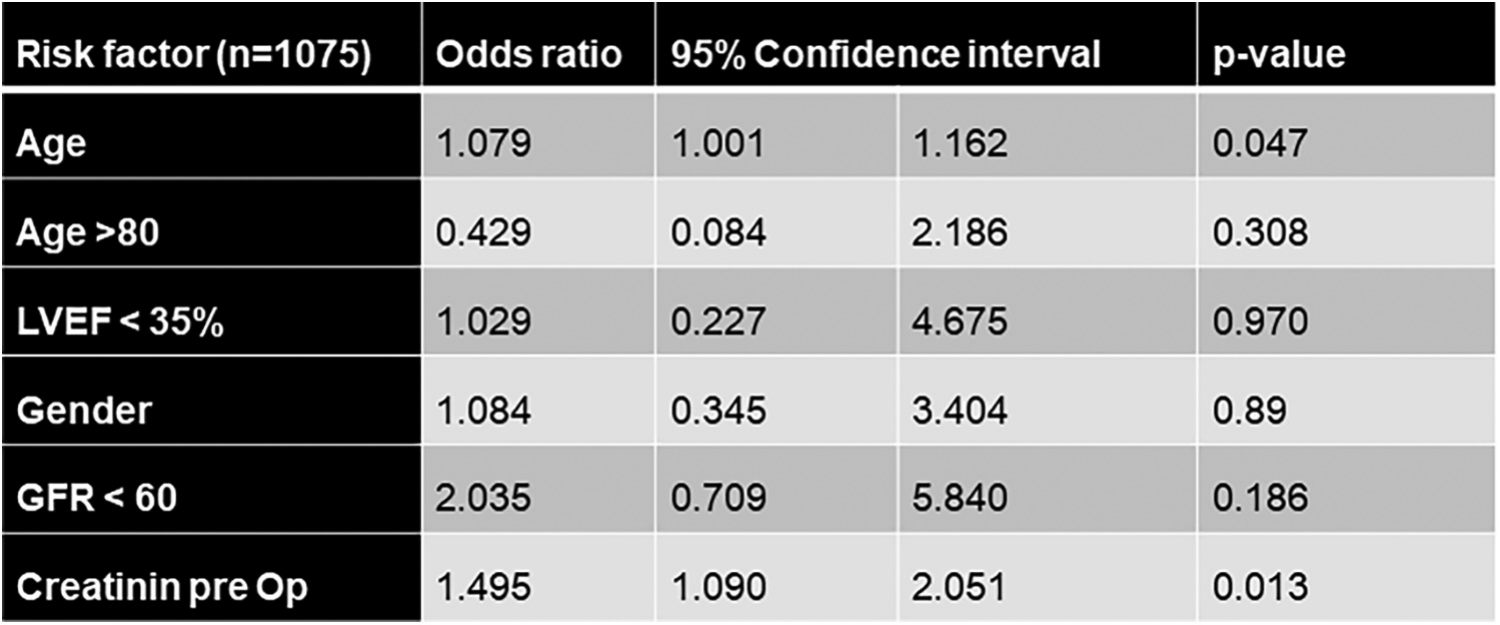
Logistic regression analysis for 30-day mortality after OPCAB. OPCAB off-pump coronary artery bypass, NYHA New York Heart Association, LVEF left ventricular ejection fraction.

Detailed results of logistic regression analysis are shown in Figure 2.

**Figure 3:**
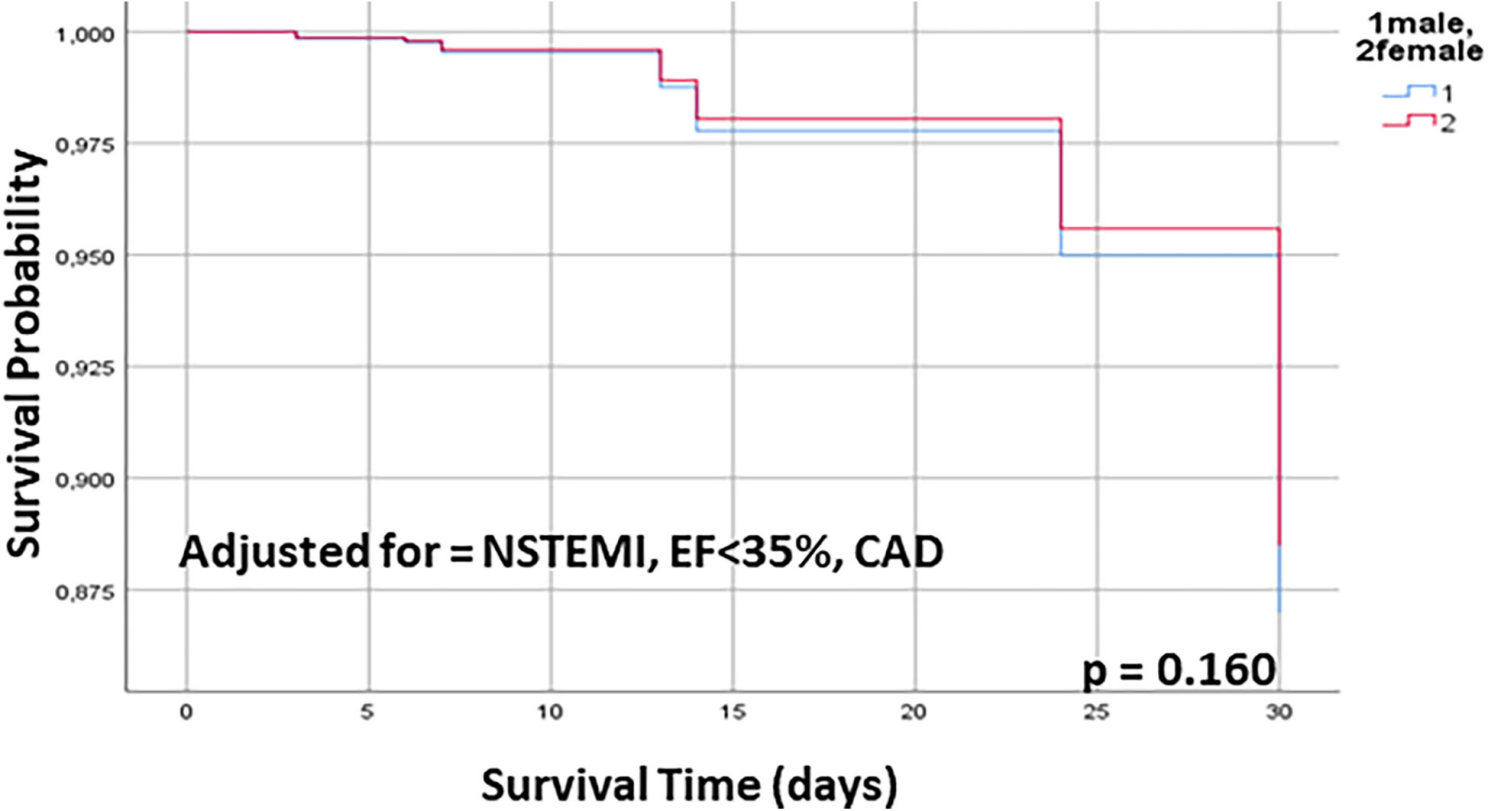
30 days Kaplan–Meier survival curve for male and female patients undergoing OPCAB, adjusted for NSTEMI, EF < 35% and extent of CAD. OPCAB off pump coronary artery bypass, NSTEMI non ST-elevation infarct, EF ejection fraction, CAD coronary artery disease.

After adjustment for rates of NSTEMI, reduced LVEF and extent of CAD no significant differences for 30-day mortality were found (Figure 3).

## Discussion

Main findings of the herein conducted study are: (I) male and female patients present similar 30-day outcomes subsequent to OPCAB regarding 30-day mortality, rates of disabling stroke, myocardial infarction and acute renal failure, (II) periprocedurally female patients receive more often and higher numbers of RBC units and rate of BIMA utilization is lower in female patients, (III) 30-day survival after OPCAB is similar between male and female patients even after adjustment for preprocedural NSTEMI, severely reduced LVEF and extent of CAD, (IV) identified risk factors for adverse 30-day outcomes after OPCAB consist of age and preoperative impaired renal function, whereas gender presented no impact on 30-day mortality after OPCAB.

Female patients are prone to impaired postoperative outcomes across a variety of surgical interventions (18) including CABG (19) with documented higher rates of mortality and even after percutaneous coronary intervention for ischemic heart disease (20). While this phenomenon is likely multi-factorial, investigation of different operative strategies in CABG is lacking and herein similar results of OPCAB in male and female patients were shown for 30-day outcomes. Although larger scale randomized controlled trials could not prove significant impact of the OPCAB approach on clinical endpoints (21), OPCAB is widely considered to present advantages in specific subsets of patients including elderly patients, patients with significant comorbidities and patients with reduced LVEF and/or diabetes (22). The herein presented results suggest that female patients might also benefit from OPCAB. Given the possible reasons for worse outcomes in female patients after CABG, which consist of a higher prevalence of microvascular disease compared to men, smaller coronary artery diameters with subsequent higher rates of graft to target vessel size mismatch and a worse preoperative status (23–25), as reflected in this work by a higher EuroSCORE II in women, OPCAB might outplay its specific advantages in women by reducing systemic inflammatory response, organ dysfunction and coagulation disorders, as well as addressing the mentioned gender-specific anatomical (small artery diameters, microvascular disease) and clinical challenges by avoiding CPB (26). This may be especially true for OPCAB procedures using total arterial revascularization and a non-aortic touch approach, which was shown to reduce rates of periprocedural stroke rates and long-term rates of re-revascularization and myocardial infarction (27, 28). Although rate of BIMA utilization in women was lower compared to male patients in our work, which may be partly attributable to higher rates of three vessel CAD in male patients, BIMA utilization rate was still markedly higher than the average of western countries (29). Acute mortality, stroke and myocardial infarction rates were not only similar between men and women in this study, but low for the entire patient cohort. These results are in line with previous findings of a retrospective study of Puskas et al., who showed decreased rates of cardiovascular events and mortality after OPCAB compared to CABG as well as decrease of those rates in women undergoing OPCAB (30). Therefore, this work adds to the growing evidence for benefits of OCPAB in women. Additionally, our findings regarding similar mortality rates between male and female patients undergoing OPCAB were consistent after adjustment for several confounders suggesting a significant advantageous impact of the OPCAB approach for myocardial revascularization in women. However, it has to be emphasized that the compared groups presented with certain differences in baseline characteristics which may be an indicator of selection bias, potentially hampering interpretability of results. The comparably high rates of BIMA utilization in women in this work may contribute to a lasting protective effect regarding mortality, re-revascularization and myocardial infarction even in the long term, since total arterial revascularization was shown to be beneficial compared to utilization of SVG (27), although this remains speculative since no long-term data are available in the context of this study. Since a long-term benefit for OPCAB compared to CABG was not documented so far (31), further studies regarding gender specific long-term effects of the surgical approach in myocardial revascularization are warranted.

The increased rates of RBC administration is still a matter of concern in women, since it was shown to be connected with increased long-term mortality (32). However, OPCAB is commonly considered to be associated with lower rates and decreased numbers of RBC administration, which might be an additional benefit of OPCAB in women. Previous work showed that female patients undergoing CABG/OPCAB tend to present older and with a higher symptom burden at time of surgery compared to male counterparts (33, 34). A phenomenon which was not confirmed by the herein presented data. Reasons for that discrepancy remain speculative but may involve an increase in awareness of gender-specific variability of CAD associated symptoms over the last decade. While age is commonly considered a risk factor for adverse outcomes in a variety of surgical interventions, and was also shown to be predictive for 30-day mortality in logistic regression analysis in this work, an impaired renal function was primarily shown to increase duration of hospital stay and costs (35) in CABG procedures. However, specific analyses regarding influence of an impaired renal function on postoperative outomes in CABG presented adverse long-term outomes and also an increase in early mortality (36) which was confirmed by the herein conducted analyses.

## Limitations

Limitations are inherent in the retrospective, single-center study design with limited patient numbers: patients were not randomized to a specific treatment, therefore patient preselection with hidden confounders may apply. Furthermore, no long-term outcomes of the herein investigated patient population is available. Female patients in this work were rather higher age and therefore post-menopausal, comparability to other studies comparing outcomes in male and female patients of younger age is therefore limited.

## Conclusions

Male and female patients present similar 30-day outcomes after OPCAB regarding mortality, stroke, myocardial infarction and renal failure suggesting a potential benefit of OPCAB in female patients. However, female patients receive more saphenous vein grafts compared to men, which may lead to impaired long-term outcomes. Further larger scale studies are warranted to clarify the impact of the surgical approach in CABG on gender specific outcomes.

## Data Availability

The raw data supporting the conclusions of this article will be made available by the authors, without undue reservation.
